# The combination of fatty liver and increased gamma-glutamyl transpeptidase levels as a risk factor for atherosclerotic plaque development in apparently healthy people

**DOI:** 10.3906/sag-1901-166

**Published:** 2019-10-24

**Authors:** Kaori KINOSHITA, Nobuyuki TOSHIKUNI, Takashi SAITO, Nobuhiko HAYASHI, Takahiro MINATO, Yasuhiro MATSUE, Yoshimichi UEDA, Mutsumi TSUCHISHIMA, Mikihiro TSUTSUMI

**Affiliations:** 1 Department of Hepatology, Kanazawa Medical University, Ishikawa Japan; 2 Department of Pathology II, Kanazawa Medical University, Ishikawa Japan

**Keywords:** Fatty liver, serum gamma-glutamyl transpeptidase, carotid plaque, apparently healthy people

## Abstract

**Background/aim:**

To elucidate how the combination of fatty liver and increased serum gamma-glutamyl transpeptidase (GGT) levels influences atherosclerotic plaque development in apparently healthy people.

**Materials and methods:**

The study population included people who had received an annual health checkup for more than 7 years and had no evidence of carotid plaque at baseline. We investigated the risk factors for carotid plaque occurrence using the Cox proportional hazards model.

**Results:**

A total of 107 people (76 men and 31 women; median age, 49 years) were enrolled. At baseline, fatty liver and a serum GGT level ≥50 U/L were observed in 13 and 38 people, respectively. During a median follow-up period of 13.3 years, carotid plaques appeared in 34 people. Multivariate analysis revealed that the combination of fatty liver and a serum GGT level ≥50 U/L was the only significant risk factor for carotid plaque occurrence (age- and sex-adjusted hazard ratio: 5.55; 95% confidence interval 1.70–18.14; P = 0.005).

**Conclusion:**

The combination of fatty liver and increased serum GGT levels raises the risk for atherosclerotic plaque development in apparently healthy people.

## 1. Introduction

Atherosclerosis is a key pathological mechanism underlying cardiovascular disease (CVD) [1]. Recent studies have provided evidence of the pivotal role of gamma-glutamyl transpeptidase (GGT) in atherosclerosis progression [2–4]. Histochemical studies revealed the presence of GGT activity in human coronary plaques [2,3]. Human carotid plaques contain glutathione, cysteinyl-glycine, cysteine, and low-density lipoprotein (LDL)/GGT complexes [4]. A possible underlying mechanism of atherosclerotic plaque development is as follows: elevated serum GGT levels may augment GGT-lipoprotein complex entry into arterial plaques, which can enhance GGT-mediated glutathione degradation and thus increase the amount of cysteinyl-glycine in the plaques. Cysteinyl-glycine has the potential to promote LDL oxidation by generating superoxide radicals via an iron reduction reaction and thus contributes to atherosclerotic plaque development [5]. A recent large-scale population-based study demonstrated that serum GGT levels are a biomarker that can predict stroke risk [6]. Furthermore, many studies have suggested the positive association of fatty liver disease with atherosclerosis [7–9]. A metaanalysis showed a strong association between nonalcoholic fatty liver disease (NAFLD) and carotid intima-media thickness and plaques [7]. Some studies have shown that NAFLD induces atherosclerosis-associated factors, including proinflammatory cytokines and coagulation factors [10,11]. Considering these data, we hypothesized that the combination of fatty liver and increased serum GGT levels may accelerate atherosclerotic plaque development. We then performed a cross-sectional study to test this hypothesis. Although our study demonstrated a close relationship between these two conditions and carotid plaque presence, the study design could not prove causality between them [12]. The aim of this longitudinal study was to determine whether the combination of these conditions raises the risk for atherosclerotic plaque development in apparently healthy people.

## 2. Materials and methods

### 2.1. Study subjects

This retrospective longitudinal study included people who had received an annual health checkup for more than 7 years at our health evaluation center and had no evidence of carotid plaque at baseline. The exclusion criteria were as follows: a history of CVD; hepatitis B or C virus infection; and medications capable of influencing the course of atherosclerosis, including antihypertensive agents, antidyslipidemic agents, antidiabetic agents, and antiplatelet/anticoagulant agents. People who had any exclusion criteria through the end of this study were excluded. The study period was between January 2001 and December 2016. The study protocol was approved by the ethics committee of Kanazawa Medical University (approval no. 144) and was conducted in accordance with the Declaration of Helsinki. Written informed consent was waived by the ethics committee.

### 2.2. Examinations

The physical examination included height, weight, and blood pressure, which was measured using a mercury sphygmomanometer. Hypertension was defined according to the Japanese Society of Hypertension guidelines for managing hypertension: systolic blood pressure ≥140 mmHg or diastolic blood pressure ≥90 mmHg [13]. Laboratory tests included serum lipid levels, such as serum triglyceride levels, measured using enzymatic color tests (Kyowa Medex Co., Ltd., Tokyo, Japan); serum high-density lipoprotein (HDL) and LDL cholesterol levels, measured using the direct method (Sekisui Medical Co., Ltd., Tokyo, Japan); fasting blood glucose levels, assessed by the hexokinase method (Shino-Test Corporation, Tokyo, Japan); serum glycated hemoglobin (HbA1c) levels, measured by an enzymatic assay (Kyowa Medex Co., Ltd., Tokyo, Japan); and serum hepatobiliary enzyme levels, such as serum aspartate aminotransferase (AST), alanine aminotransferase (ALT), and GGT levels, measured using the Japan Society of Clinical Chemistry (JSCC) standardization method (Kanto Chemical Co., Inc., Tokyo, Japan). Smoking status was classified as follows: never smoked, past smoking, and current smoking. The alcohol consumption questionnaire assessed the number of drinking days/week over a period of at least one year, and alcohol consumption was evaluated according to the number of standard drinks/drinking day (one standard drink is defined as 10 g of alcohol in Japan). Thus, average daily alcohol consumption was calculated as follows: (number of drinking days/week) × (alcohol consumption/drinking day)/7.

Carotid and abdominal ultrasound (US) examinations were routinely performed in a health checkup at our health evaluation center. Experienced ultrasonographers who were blinded to the health checkup data of each person performed these examinations. High-resolution US machines (Aplio XG/500/80, Canon Medical Systems Corporation, Tochigi, Japan) and an 8.4- or 9-MHz probe were used for carotid US. Carotid plaques were defined as intima-media thickness >1.5 mm in any portion of the carotid arteries [14]. The same US machines and a 5-MHz probe were used for abdominal US. The diagnosis of fatty liver was made on the basis of increased echogenicity of the liver parenchyma compared to the echogenicity of the right renal cortex [15].

### 2.3. Statistical analysis

Continuous variables are expressed as the median (25th, 75th percentile). We calculated the cumulative incidence rates of carotid plaque occurrence with the Kaplan–Meier method and performed log-rank tests to evaluate the rate differences. We investigated the risk factors for carotid plaque occurrence using the Cox proportional hazards regression model. The following variables were potential risk factors: age (years), sex, body mass index (BMI; kg/m2), hypertension (absence/presence), serum triglyceride level (mg/dL), serum HDL cholesterol level (mg/dL), serum LDL cholesterol level (mg/dL), fasting blood glucose level (mg/dL), serum HbA1c (%), serum GGT level (U/L), fatty liver (absence/presence), smoking status (never smoked/past and current smoking), and alcohol consumption (≤20 g/day/>20, <60 g/day/≥60 g/day). We created two statistical models. The first model used the variables described above (model 1), and the second model used the following combinations of the presence or absence of fatty liver and serum GGT levels (a mean value of 50 U/L was the cut-off): nonfatty liver and a serum GGT level <50 U/L, fatty liver and a serum GGT level <50 U/L, nonfatty liver and a serum GGT level ≥50 U/L, and fatty liver and a serum GGT level ≥50 U/L (model 2). We performed a multivariate analysis using variables with P < 0.2, according to a univariate analysis, and calculated the age- and sex-adjusted hazard ratios of the variables, and P < 0.05 was considered statistically significant. All statistical analyses were performed using STATA version 13.1 (STATA Corp., College Station, TX, USA).

## 3. Results

### 3.1. Baseline characteristics of the cohort

A total of 599 people received a health checkup annually for more than 7 years. However, 492 people received medications capable of influencing the course of atherosclerosis before the study and/or during the study period. Thus, this study enrolled 107 people (76 men and 31 women; median age, 49 years). At baseline, fatty liver and a serum GGT level ≥50 U/L were observed in 13 and 38 people, respectively (Table 1). 

**Table 1:** Baseline characteristics of the whole cohort (n = 107).

Variable	
Age, years	49 (44, 55)
Sex, men/women	76/31
BMI, kg/m2	22.5 (21.1, 24.8)
Hypertension, no/yes	103/4
Triglyceride, mg/dL	104 (75, 159)
HDL cholesterol, mg/dL	56 (47, 67)
LDL cholesterol, mg/dL	121 (98, 141)
FBG, mg/dL	91 (86, 97)
HbA1c, %	5.5 (5.2, 5.8)
AST, U/L	22 (19, 26)
ALT, U/L	22 (16, 31)
GGT, U/L	31 (19, 72)
GGT ≥50 U/L, no/yes	69/38
Fatty liver, no/yes	94/13
Smoking, never/past/current	46/27/34
Drinking, <20/>20, <60/≥60, g/day	84/6/17

Continuous variables are expressed as medians (25th, 75th percentile). BMI: body mass index; HDL: high-density lipo-protein; LDL: low-density lipoprotein; FBG: fasting blood glucose; HbA1c: serum glycated hemoglobin; AST: aspartate aminotransferase; ALT: alanine aminotransferase; GGT: gam-ma-glutamyl transpeptidase.

### 3.2. Cumulative incidence rates of carotid plaque occurrence

**Figure 1:**
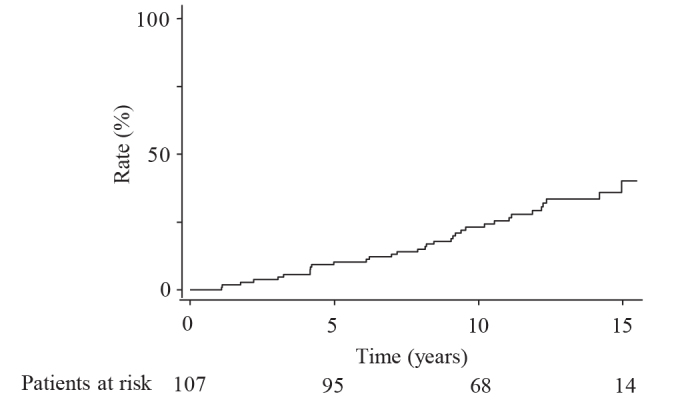
Incidence rates of carotid plaque occurrence in the whole cohort (n = 107).

**Figure 2:**
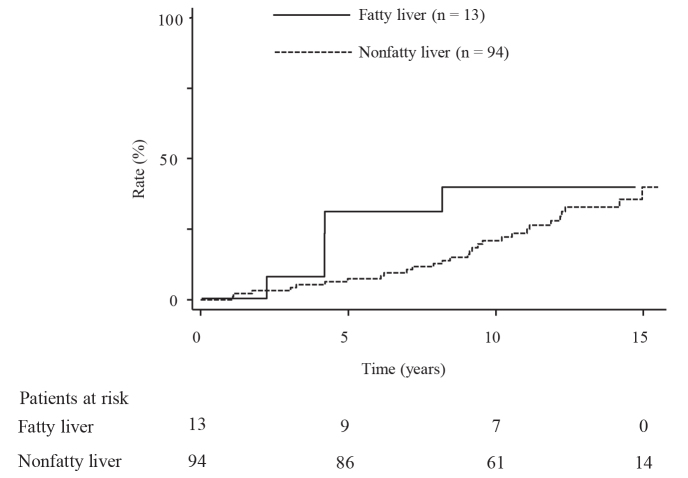
Comparison of the incidence rates of carotid plaque occurrence between people with and without fatty liver (P = 0.38).

**Figure 3:**
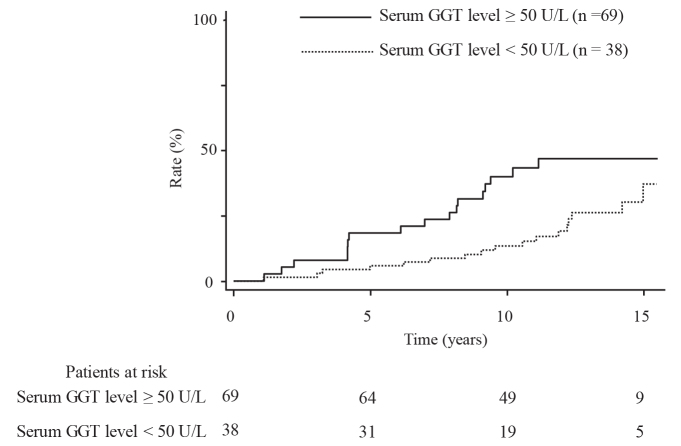
Comparison of the incidence rates of carotid plaque occurrence between two groups divided according to serum GGT levels (P = 0.020).

**Figure 4:**
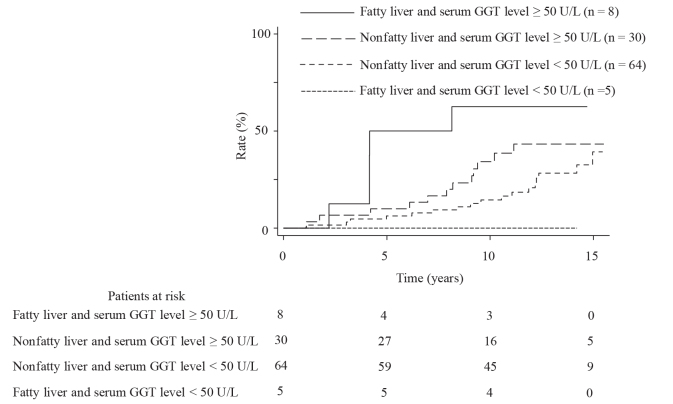
Comparison of the incidence rates of carotid plaque occurrence among four groups divided according to the presence or absence of fatty liver and serum GGT levels (P = 0.017).

All the enrolled participants received the carotid and abdominal US examinations annually. During a median follow-up period of 13.3 years (range: 7.2 to 15.5 years), carotid plaques emerged in 34 people (24 men and 10 women); the cumulative incidence rates were 10%, 23%, and 40% at 5, 10, and 15 years, respectively (Figure 1). There was no significant difference in the incidence rates of carotid plaque between people with or without fatty liver (P = 0.38) (Figure 2). However, the incidence rates were significantly higher in people with a serum GGT level ≥50 U/L than in those with a serum GGT level <50 U/L (P = 0.020) (Figure 3). When the subjects are divided into four groups according to the presence or absence of fatty liver and serum GGT levels, those with fatty liver and a serum GGT level ≥50 U/L (n = 8) had the highest incidence rates, followed by those with only a serum GGT level ≥50 U/L (n = 30), those without the presence of either factor (n = 64), and those with fatty liver only (n = 5) (P = 0.017); the rates for these groups at 10 years were 62.5%, 34.3%, 14.5%, and 0%, respectively (Figure 4).

**Table 2:** Risk factors for carotid plaque occurrence (model 1).

	Univariate analysis			Multivariate analysis		
Variable	HR	95% CI	P-value	HR	95% CI	P-value
Age, years	1.00	0.96–1.04	0.90	0.99	0.94–1.03	0.51
Men	0.97	0.46–2.03	0.94	0.89	0.41–1.94	0.77
BMI, kg/m2	1.08	0.96–1.21	0.22			
Hypertension	0.75	0.10–5.50	0.78			
Triglyceride, mg/dL	1.001	0.999–1.002	0.099	1.00	0.99–1.01	0.90
HDL, mg/dL	0.97	0.95–0.99	0.025	0.98	0.95–1.01	0.15
LDL, mg/dL	1.01	0.99–1.02	0.14	1.01	1.00–1.02	0.18
FBG, mg/dL	1.00	0.96–1.03	0.83			
HbA1c, %	0.95	0.36–2.46	0.91			
GGT, U/L	1.006	1.001– 1.012	0.021	1.005	0.999–1.011	0.11
Fatty liver	1.53	0.59–3.97	0.38			
Never smoked	1.00					
Past and current smoking	2.56	1.19–5.50	0.016	1.81	0.79–4.14	0.16
Drinking ≤20 g/day	1.00					
Drinking >20, <60 g/day	1.09	0.26–4.62	0.90			
Drinking ≥60 g/day	1.52	0.66–3.51	0.33			

HR: hazard ratio; CI: confidence interval; BMI: body mass index; HDL: high-density lipo-protein; LDL: low-density lipoprotein; FBG: fasting blood glucose; HbA1c: serum glycated hemoglobin; GGT: gamma-glutamyl transpeptidase.

**Table 3:** Risk factors for carotid plaque occurrence (model 2).

	Univariate analysis			Multivariate analysis		
Variable	HR	95% CI	P-value	HR	95% CI	P-value
Age, years	1.00	0.96–1.04	0.90	0.98	0.94–1.03	0.41
Men	0.97	0.46–2.03	0.94	0.78	0.35–1.71	0.53
BMI, kg/m2	1.08	0.96–1.21	0.22			
Hypertension	0.75	0.10–5.50	0.78			
Triglyceride, mg/dL	1.001	0.999–1.002	0.099	1.00	0.99–1.01	0.59
HDL, mg/dL	0.97	0.95–0.99	0.025	0.97	0.94–1.00	0.053
LDL, mg/dL	1.01	0.99–1.02	0.14	1.01	1.00–1.02	0.25
FBG, mg/dL	1.00	0.96–1.03	0.83			
HbA1c, %	0.95	0.36–2.46	0.91			
Nonfatty liver and GGT <50 U/L	1.00					
Fatty liver and GGT <50 U/L	NA	NA	NA	NA	NA	NA
Nonfatty liver and GGT ≥50 U/L	1.69	0.81–3.55	0.16	1.56	0.69–3.54	0.29
Fatty liver and GGT ≥50 U/L	3.78	1.38–10.31	0.009	5.55	1.70–18.14	0.005
Never smoked	1.00					
Past and current smoking	2.56	1.19–5.50	0.016	1.74	0.76–4.03	0.19
Drinking ≤20 g/day	1.00					
Drinking >20, <60 g/day	1.09	0.26–4.62	0.90			
Drinking ≥60 g/day	1.52	0.66–3.51	0.33			

HR: hazard ratio; CI: confidence interval; BMI: body mass index; HDL: high-density lipoprotein; LDL: low-density lipoprotein; FBG: fasting blood glucose; HbA1c: serum glycated hemoglobin; GGT: gamma-glutamyl transpeptidase; NA: not available.

### 3.3. Risk factors for carotid plaque occurrence (model 1)

We first performed a Cox proportional hazards regression analysis using the variables in model 1. Univariate analysis revealed that the variables with P < 0.2 were serum triglyceride level, serum HDL cholesterol level, serum LDL cholesterol level, serum GGT level, and past and current smoking. However, multivariate analysis identified no significant predictors (Table 2).

### 3.4. Risk factors for carotid plaque occurrence (model 2)

We next performed an analysis using the variables in model 2. Univariate analysis revealed that the variables with P < 0.2 were serum triglyceride level, serum HDL cholesterol level, serum LDL cholesterol level, nonfatty liver and a serum GGT level ≥50 U/L, fatty liver and a serum GGT level ≥50 U/L, and past and current smoking. According to the multivariate analysis, the combination of fatty liver and a serum GGT level ≥50 U/L was the only significant risk factor for carotid plaque occurrence (age- and sex-adjusted hazard ratio: 5.55; 95% confidence interval: 1.70–18.14; P = 0.005) (Table 3).

## 4. Discussion

In the current study, we have demonstrated that the combination of fatty liver and increased serum GGT levels increases the risk for atherosclerotic plaque development in apparently healthy people. Our previous cross-sectional study found a close relationship between the combination of these two conditions and the presence of carotid plaques [12]. This longitudinal study clearly showed the causal relationship between them. Because many studies have demonstrated a close link between carotid atherosclerosis and future CVD events, people with both conditions are considered to be at high risk for CVD [16–18]. In clinical practice, physicians should pay attention to such people, urge them to receive a carotid US examination, and follow them closely.

The current study revealed a novel biomarker for predicting atherosclerotic plaque development in apparently healthy people. In planning this study, we suspected that it would be difficult to precisely estimate the impacts of medications capable of influencing the course of atherosclerosis. Therefore, we excluded people who received such medications in the past and would be taking them during the study period. As a result, apparently healthy people were enrolled as the study subjects. Many epidemiological studies have reported risk factors for atherosclerosis development, including hypertension, dyslipidemia, diabetes mellitus, obesity, and smoking [19]. Indeed, low serum HDL cholesterol levels, past and current smoking, and the combination of fatty liver and increased serum GGT levels were significant factors according to our univariate analysis. However, in the multivariate analysis, only the combination of the two conditions remained significant. Assessing these conditions may be useful for identifying people at high risk for atherosclerotic plaque development in apparently healthy populations.

The results of the current study are consistent with those of a recent large-scale longitudinal study that investigated the causal relationship between NAFLD and carotid atherosclerosis [8]. This recent study revealed that persistent NAFLD is a significant risk factor for carotid atherosclerosis, and that persistently high serum GGT levels in people with NAFLD increase the risk of carotid atherosclerosis development. Nevertheless, our study has some advantages. First, the median follow-up period was much longer in our study than that in the other study (13.3 years vs. 3.3 years). Second, the people who received medications capable of influencing the course of atherosclerosis were excluded from our study. Thus, the impacts of fatty liver and serum GGT levels on atherosclerosis progression were more directly shown.

Our results suggest that not all fatty livers can exert a promoting effect on plaque development. When dividing people into four groups according to the presence or absence of fatty liver and serum GGT levels, people with both fatty liver and a serum GGT level ≥50 U/L had the highest incidence rates of carotid plaque occurrence. In contrast, people with fatty liver and a serum GGT level <50 U/L did not develop carotid plaques. Recent epidemiological studies have found that several factors, such as alcohol consumption, smoking, aging, and obesity can increase serum GGT levels [20–23]. Regarding such factors, there were no significant differences between people with fatty liver and a serum GGT level ≥50 U/L and those with fatty liver and a serum GGT level <50 U/L (data not shown). Other recent studies have demonstrated that patients with advanced fatty liver disease have high serum GGT levels [24]. We speculate that our subjects with fatty liver and high serum GGT levels may have advanced fatty liver disease. Future studies will be required to test this hypothesis.

The current study has some limitations. First, this study had a small number of subjects, which may have reduced its statistical power. Second, fatty liver was assessed by routine US examinations. The low diagnostic accuracy in assessing mild fatty liver with US may have affected the study results [15]. However, recent US technologies have improved accuracy for fatty liver diagnosis [25]. Future studies should be performed with advanced technologies. Third, this study included only Japanese subjects, which may limit the generalizability of the study results.

In conclusion, our results demonstrate that the combination of fatty liver and increased serum GGT levels increases the risk for atherosclerotic plaque development. Assessing these two conditions can help identify people who are at high risk of future CVD among apparently healthy populations.

## Acknowledgements

This work was supported by a grant for specially promoted research from Kanazawa Medical University (SR2012-04) and a grant from St Luke’s Life Science Institute (Tokyo, Japan).
